# Characterization of the family of Mistic homologues

**DOI:** 10.1186/1472-6807-6-10

**Published:** 2006-05-16

**Authors:** Tarmo P Roosild, Mark Vega, Samantha Castronovo, Senyon Choe

**Affiliations:** 1 Structural Biology Laboratory, The Salk Institute for Biological Studies, La Jolla, California 92037, USA

## Abstract

**Background:**

Mistic is a unique *Bacillus subtilis *protein with virtually no detectable homologues in GenBank, which appears to integrate into the bacterial membrane despite an overall hydrophilic composition. These unusual properties have been shown to be useful for high-yield recombinant expression of other membrane proteins through fusion to the C-terminus of Mistic. To better understand the structure and function of Mistic, we systematically searched for and characterized homologous proteins among closely related bacteria.

**Results:**

Three homologues of Mistic were found with 62% to 93% residue identity, all only 84 residues in length, corresponding to the C-terminal residues of *B. subtilis *Mistic. In every case, the Mistic gene was found partially overlapping a downstream gene for a K^+ ^channel protein. Residue variation amongst these sequences is restricted to loop regions of the protein's structure, suggesting that secondary structure elements and overall fold have been conserved. Additionally, all three homologues retain the functional ability to chaperone fusion partners to the membrane.

**Conclusion:**

The functional core of Mistic consists of 84 moderately conserved residues that are sufficient for membrane targeting and integration. Understanding the minimal structural and chemical complexity of Mistic will lead to insights into the mechanistic underpinnings of Mistic-chaperoned membrane integration, as well as how to optimize its use for the recombinant heterologous expression of other integral membrane proteins of interest.

## Background

Integral membrane (IM) proteins constitute nearly a third of the proteins of sequenced genomes and play critical roles in intercellular signaling, homeostasis and metabolite transport. Additionally, they are the target of a majority of therapeutic pharmaceuticals. However, our understanding of this class of proteins has lagged that of soluble proteins due to inherent difficulties in their recombinant production and their structural analysis. A new method to overcome the first obstacle recently emerged with the discovery of Mistic, a unique hydrophilic protein from *Bacillus subtilis *that associates with the bacterial membrane, and when fused to the N-terminus of other IM proteins can chaperone their expression in *E. coli *at high yields [1]. It has been proposed that Mistic is able to autonomously integrate, in a Sec-independent manner, into the lipid bilayer. This is based on the indirect evidence that the protein lacks a stretch of hydrophobic amino acids that could be interpreted, mechanistically, as a signal sequence by the bacteria's translocon machinery. Additionally, high level expression of Mistic and Mistic fusions can be achieved without the toxicity normally observed with recombinant expression of IM proteins at levels saturating the secretory system. More recently it was shown that Mistic fused to GFP partitioned to liposomes in a cell-free expression system lacking a translocon system [2]. Nevertheless, the physical mechanism by which Mistic accomplishes its chaperoning function remains unclear, and it is almost imperative to hypothesize that the highly hydrophilic surface of the NMR structure of Mistic must undergo a substantial, dynamic, conformational transition in order to associate with the membrane.

**Figure 1:**
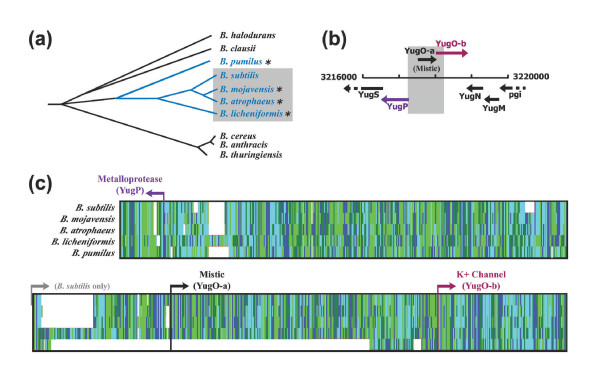
**Phylogenetic distribution and genetic structure of Mistic**. (a) Phylogenetic tree showing the relationship between the Bacillus species probed in this study (starred) or those with fully sequenced genomes. Those with the YugP (metalloprotease) and YugO-b (K+ channel) genes sequentially arranged in their chromosome are indicated in blue. Those with Mistic homologues are highlighted (grey). (b) Genetic structure of the region in *B. subtilis *analyzed by this study, with the sequenced region between YugP (purple) and YugO-b (magenta) highlighted (grey). (c) Sequence alignment of the genetic material between the YugP and YugO-b genes from five Bacillus species (A = green, C = teal, G = blue, T = lime) highlighting deletions and insertions (white). Translation initiation codons are marked with arrows, colored as in (b). *B. pumilus *has approximately 250 fewer bases between the two primary genes, consistent with the lack (or loss) of an 84 residue protein preceding the K+ channel open-reading-frame. The genomic region of *B. subtilis *that encodes the first 26 amino acids of its Mistic homologue (between the grey and black arrows), aligns with numerous frame-shifting mutations and stop codons (not shown) in even the most closely related species (*B. mojavensis*) suggestive that the internal methionine (residue 27) may also serve as an *in vivo *translation initiation start site for Mistic.

To obtain better understanding of protein structure-function relationships, one can examine residue conservation patterns among homologous family members in a class of proteins. These patterns have been used to identify residues critical for protein structure and folding [3], demonstrate ligand and substrate specificity [4], determine active site catalytic residues [5], identify protein-protein interaction interfaces [6], and uncover allosteric modulation pathways through a protein domain [7,8]. The application of this approach to Mistic, however, has been hampered by the complete absence, until very recently with the addition of *B. licheniformis*, of any proteins with detectable homology to Mistic using conventional Blast algorithms against the public genome database (GenBank), despite the presence of the genomes of *B. anthracis, B. cereus, B. thuringiensis, B. halodurans*, and *B. clausii *(Figure 1a). To overcome this limitation, we systematically probed Bacillus species closely related to *B. subtilis *to determine the natural distribution of this gene and to find homologous proteins for comparative analysis. Our results reveal a protein core for Mistic, consisting of the C-terminal 84 residues, that is conserved amongst the evolutionarily-nearest neighbors of *B. subtilis *and is sufficient to chaperone recombinant IM proteins to the lipid bilayer.

## Results and discussion

### Mistic gene structure

Genomic DNA from four species of Bacillus closely related to *B. subtilis *was amplified using two 'MisticSeeker' oligonucleotides complementary to conserved regions of the upstream gene, YugP, and the downstream gene, YugO-b (Figure 1b). For *B. licheniformis, B. mojavensis *and *B. atrophaeus*, amplified PCR fragments produced nucleotide sequence data of 900–950 base pairs, similar to that expected based on the positioning of the MisticSeeker primers with respect to the *B. subtilis *genome. In contrast, the primers only produced a 651 base pair fragment from *B. pumilus *genomic DNA. Alignment of the base sequences reveals general conservation of this region of the Bacillus chromosome, with higher degrees of conservation within the predicted open-reading-frames of the flanking genes, as expected. Of the variations observed, most notable is that *B. pumilus *has a large deletion (~250 bps) in the region encoding Mistic in *B. subtilis *(Figure 1c). Also, while there are no gaps or insertions in the 252 base pairs that encode for the C-terminal 84 residues of Mistic in *B. subtilis*, there are ample such frame shifting mutations in the region that aligns with the coding region for the first 26 residues of the original Mistic. Our data for *B. licheniformis *are consistent with that recently published for its sequenced genome [9,10].

### Mistic homologues have only 84 residues

**Figure 2:**
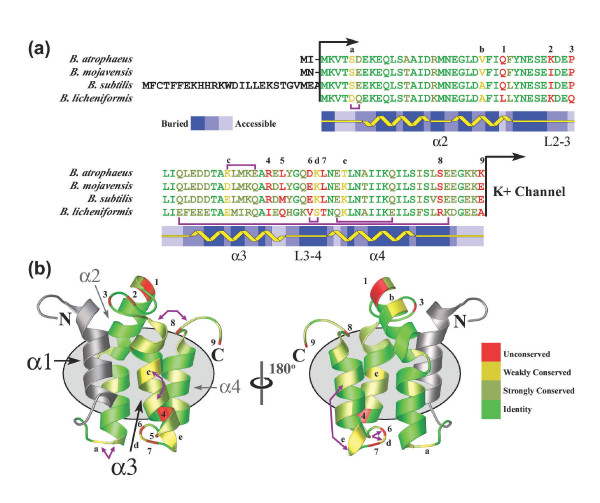
**Sequence and structure of Mistic homologues**. (a) Sequence alignment of the four 84-residue Mistic homologues is shown, as well as the leading 26 amino acids found only in *B. subtilis *Mistic. Residues are colored by their degree of conservation, as indicated. Below the alignment, secondary structural elements are depicted as well as residue accessibility. Pairs of compensating mutations related to conservation of acidic residues are indicated by purple brackets. (b) Unconserved residues (numbered 1–9) and weakly conserved residues (a-e) are mapped to the NMR structure of Mistic (colored as above) revealing their restriction to flexible, loop regions of the protein. The core of the structure (grey oval) is virtually devoid of significant alterations. This pattern suggests that the overall structural fold of the protein, less the N-terminal helix (α1, grey), is conserved between the four homologues. Positions of compensating mutational pairs for anionic residues are indicated by purple arrows revealing their close proximity in the folded protein.

Conceptual translation of the DNA sequences from *B. licheniformis, B. mojavensis *and *B. atrophaeus *revealed 252 base pair open-reading-frames that translated to proteins homologous to the C-terminal 84 amino acids of the original *B. subtilis *Mistic protein (Figure 2a). In all three cases, the Mistic homologue is located just upstream and partially overlapping (by four base pairs, including the stop codon for Mistic) the K^+ ^channel gene (YugO-b). For *B. pumilus*, no open-reading frame was found in the sequence determined for the amplified region between YugP and YugO-b. The percent identity residue conservation for these homologues varied from 93% to 62% for the 84 amino acid core region of the protein and correlated with the evolutionary distance between species. Interestingly, *B. subtilis *Mistic possesses an internal methionine at its 27^th ^residue, equivalent to the first residue in all of the homologous proteins, as well as a reasonable upstream ribosome binding site, suggestive of an alternative internal translation initation for this protein that might also produce an 84-residue variant of Mistic. The resulting truncated variant of Mistic from *B. subtilis *is remarkably less chemically diverse due to loss of its only cysteine and tryptophan residues as well as three of its four phenylalanines. Surprisingly, it is also more hydrophilic and acidic with fully one quarter of its residues either aspartate or glutamate.

### Mistic homologues conserve structurally intrinsic and acidic residues

The degree of conservation of a residue (determined as described in Methods) was mapped to the NMR structure of Mistic (PDB id: 1YGM). Unconserved residues are generally restricted to loop regions or the C-terminus of Mistic leaving a subset of highly conserved residues forming the core of the Mistic structure (Figure 2b). This pattern is consistent with the retention of secondary structural elements, as well as the overall protein fold, amongst the homologues. The first forty residues of Mistic are significantly more conserved (30 of 40 strictly conserved) than the C-terminal half (15 of 44). This aspect of the conservation pattern is even more striking in the comparison of the two interhelical loops: L2-3 has 9 of 13 residues strictly conserved, whereas L3-4 only retains a single glycine out of seven residues. Perhaps not coincidentally, the N-terminal 40 residues also contain the two most prominently distinct structural elements of Mistic, namely a substantial kink located at the centre of the longest helix (α2) and a partially re-entrant loop (L2-3) that buries several consecutive residues in the core of the helical bundle. These elements may play critical roles in modulating Mistic's conformational changes. Buried residues are conserved to a greater degree than accessible residues (27 of 41 vs. 18 of 43) again supporting the notion that the interhelical interactions that stabilize the folded bundle are retained within the family of Mistic proteins. Most intriguing however, is the nearly strict conservation of the distribution of acidic residues (aspartate, glutamate) over the surface of Mistic's structure. Discounting the three highly flexible C-terminal residues, there are only five cases (out of 19) where a negatively charged residue is not strictly conserved in sequence space. In each of these instances, a compensating mutation can be found structurally proximate to the site of the alteration (Figure 2). Four of these pairs move the negative charge to an adjacent residue, one or two turns along the face of a helix, or to an adjacent alternate loop, respectively, while retaining a single negative charge. In the fifth case, the compensating loss of an adjacent cationic lysine residue maintains locally a similar overall net surface charge distribution. These observations suggest that the highly anionic nature of Mistic may be central to its mechanism of targeting and associating with the lipid bilayer.

### Mistic homologues retain chaperone function

**Figure 3:**
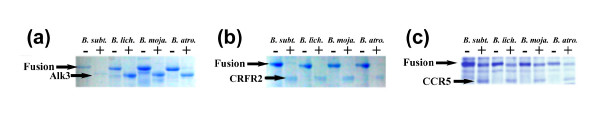
**Efficiency of Mistic homologues in promoting expression of C-terminally fused membrane proteins**. Shown are three SDS-polyacrylamide gels stained with Coomassie Blue showing the successful expression of: (a) Alk3 (a TGF-β receptor); (b) CRFR2β (a GPCR); and (c) CCR5 (a GPCR), by fusion to the C-terminus of variations of Mistic from *B. subtilis*, *B. licheniformis, B. mojavensis*, and *B. atrophaeus*. Successful cleavage the fusion protein (upper arrows) at the protease sensitive linker between a particular cargo protein and its respective Mistic domain in the presence of thrombin (columns marked "+"), confirms the identity of the desired product (lower arrows).

We tested the ability of the Mistic homologues, as well as the shorter form of Mistic from *B. subtilis*, to chaperone the expression of other IM proteins when fused to Mistic's C-terminus. Utilizing as test cases two topologically distinct cargo proteins, Alk3 (a TGF-β receptor) and CRFR2β (a GPCR), the Mistic-tagged fusion proteins were expressed and purified in parallel and yields compared. All four homologues produced roughly equivalent, high yields of both proteins based on SDS-PAGE analysis (Figure 3a–b). Surprisingly, the observed yields were similar to those obtained with the 110 amino acid variation of Mistic, indicating that the first 26 residues of Mistic are not essential to its membrane targeting function. Further, we tested the ability of these proteins to produce the GPCR CCR5, which in previous studies had yielded very low quantities of protein. With a more optimal linker between the Mistic domain and CCR5, all of these fusions could produce CCR5 in the membrane at approximately 1 mg/liter shaker culture (Figure 3c).

## Conclusion

Mistic is a protein that assists the integration into the lipid bilayer of covalently-linked, recombinantly expressed IM proteins, but the mechanisms underlying this ability are unknown. We have shown here that the functional core of Mistic consists of the C-terminal 84 residues. This core conserves to a substantial degree residues critical to the formation of helical secondary structural elements as well as residues important for interhelical interactions. Even more strictly conserved is the surface distribution of an abundance of acidic residues. While this characteristic may seem incompatible with membrane association, in many respects it chemically and structurally resembles amphiphilic, anionic fusogenic peptides like the synthetic 20-amino acid peptide with 5 glutamates derived from the amino-terminal segment of hemagglutinin of influenza virus [11,12]. Analogous functional mechanisms may also exist here, as both proteins play roles in membrane targeting of downstream proteins.

The existence of functional, truncated Mistic homologues raises the question as to what role the first helix (α1) of the four-helical bundle structure plays in Mistic's membrane association mechanism or its membrane topology. One plausible explanation is that the hydrophobic core of the Mistic bundle, which is exposed with removal of the N-terminal helix (α1), is in fact the hydrophobic surface that forms the interface for association with the lipid bilayer. This model is consistent with the previous observation that mutation of a core methionine within this putative hydrophobic surface to a more hydrophilic residue reduces Mistic's membrane affinity and chaperone efficacy [1]. In this case, Mistic's association peripherally, with the outer leaflet of the inner membrane, would force any trailing fusion protein linked to the C-terminus of Mistic to interact with and partition between the membrane and periplasmic space, thus facilitating its integration. This mode of association is consistent with the recent analysis of the structural nature of proteins at the membrane-water interface that concludes that the presence of charged, amphiphilic helices positioned interfacially, roughly parallel with the membrane surface, is not irregular [13].

Mistic's *in vivo *function is equally perplexing. We have demonstrated that the distribution of this gene is restricted to a very limited number of closely related soil Bacilli and is always linked to the bacterium's K^+ ^channel gene as overlapping reading frames. However, these observations have not yet produced a testable hypothesis as to Mistic's natural function. So far, the simple gene knockout produces no obvious phenotype (unpublished data). Nonetheless, the sequence conservation pattern observed within the family of Mistic proteins provides an additional clue towards elucidating the molecular mechanism of Mistic-facilitated membrane association, uncovering its natural function, and potentially optimizing its sequence for the recombinant heterologous expression of other IM proteins of interest.

## Methods

### Cloning of Mistic homologues

Genomic DNA was obtained from the Bacillus Genetic Stock Center (BGSC) for *B. licheniformis*, BGSCID 5A36; *B. mojavensis*, BGSCID 28A1; *B. atrophaeus*, BGSCID 11A1; *B. pumilus*, BGSCID 8A3 and was amplified using two 'MisticSeeker' oligonucleotides. (MisticSeekerOligo5':ATGCTAATACGACTCACTATAGGGGCTCTTTACTTTAAATTGTGCCC; MisticSeekerOligo3':ATGGCTAGTTATTGCTCAGCGGCCGACTGWNGANACNGTNABNABNGCCCACCADATNCC) PCR was conducted for 30 cycles with one minute incubations between melting (94°C), annealing (50°C), and elongation (72°C), temperatures using Vent DNA polymerase. The amplified product was sequenced using the same MisticSeeker oligos. Both DNA sequences and conceptually translated protein sequences were analyzed and aligned using ClustalW [14]. Residues were categorized as either having a single, fully conserved residue, being strongly conserved (STA; NEQK; NHQK; NDEQ; QHRK; MILV; MILF; HY; FYW), weakly conserved (CSA; ATV; SAG; STNK; STPA; SGND; SNDEQK; NDEQHK; NEQHRK; FVLIM; HFY), or unconserved, consistent with the positively scoring groups that occur in the Gonnet Pam250 matrix. Secondary structural boundaries and residue accessibility were calculated and drawn using PROCHECK [15].

### Expression of fusion proteins with Mistic homologues

Mistic was cloned by PCR into Gateway^® ^destination (Invitrogen) vectors for expression studies on eukaryotic IM proteins. Eukaryotic target genes in Gateway^® ^donor vectors were recombined with destination vectors to create expression vectors with the cargo protein fused downstream of Mistic with a separation linker of 19 amino acids. Freshly transformed colonies were cultured in TB and induced with 0.1 mM isopropyl-β-Dthiogalactopyranoside (IPTG) at an O.D. of 1.0. Growth was continued overnight at 10–18°C. Cells were harvested and resuspended in 50 mM Tris pH 8.0, 300 mM KCl, 10% glycerol, 10 mM imidazole with 1 mg/ml lysozyme. Cells were disrupted by sonication on ice and membranes were pelleted by high speed centrifugation (100,000 × g). Membranes were solubilized by sonication in the above buffer with the addition of 20 mM LDAO. Insoluble material was removed by high speed centrifugation and the desired protein was purified from the resulting supernatant using Ni-NTA affinity chromatography (Qiagen). Purified protein was analyzed by SDS-PAGE, before and after overnight incubation with thrombin at 4°C.

## Authors' contributions

TPR conceived the study, conducted DNA sequence analysis in *B. atrophaeus, B. mojavensis *and *B. licheniformis*, conducted subsequent sequence alignment analysis and structural mapping of conservation patterns, participated in CCR5 expression analysis, and drafted the manuscript. MV designed and created vectors for protein expression assays and conducted expression analysis of Alk3 and CRFR2β. S. Castronovo conducted DNA sequence analysis in *B. pumilus *and participated in CCR5 expression analysis. S. Choe participated in the design and coordination of the study and helped to draft the manuscript. All authors read and approved the final manuscript.
